# Treatment For Infected Frozen Elephant Trunk Prosthesis Caused by *Propionibacterium acnes*: A Surgical Challenge

**DOI:** 10.21470/1678-9741-2023-0186

**Published:** 2023-11-08

**Authors:** Laura Varela Barca, Laura Esteban-Lucia, Marta Tomás-Mallebrera, Ana Pello-Lázaro, Gonzalo Aldámiz-Echevarría

**Affiliations:** 1 Cardiovascular Surgery Department, Instituto de Investigación Sanitaria - Fundación Jiménez Diaz, Madrid, Spain; 2 Cardiology Department, Instituto de Investigación Sanitaria - Fundación Jiménez Diaz, Madrid, Spain; 3 Radiology Department, Instituto de Investigación Sanitaria - Fundación Jiménez Diaz, Madrid, Spain

**Keywords:** Aortic Valve Insufficiency, Aortic Diseases, Propionibacterium acnes, Dilatation, Blood Vessels Prosthesis Implantation, Stents

## Abstract

In this article, we present the case of a 47-year-old man who underwent Bentall-Bono procedure and frozen elephant trunk prosthesis implantation due to severe aortic regurgitation and aortic dilatation with a second-time endovascular stent-graft repair in descending aorta. Over eight years, a subacute graft infection by Propionibacterium acnes was developed, culminating in cardiogenic shock secondary to severe aortic regurgitation due to a complete aortic root dehiscence because of multiple aortic pseudoaneurysms. The patient underwent emergency surgery in which the replacement of the graft by a biological valve tube was performed accompanied by a complete debranching of the three supra-aortic vessels.

## INTRODUCTION

**Table:** 

Abbreviations, Acronyms & Symbols	
CT	= Computed tomography
HP	= Hybrid prosthesis
LCA	= Left coronary artery
RCA	= Right coronary artery
SAV	= Supra-aortic vessels
TTE	= Transthoracic echocardiogram

The rate of infection in aortic prosthetic devices is approximately 1% to 3% though these events are linked to a high rate of mortality (25-80%)^[1]^. In cases of aortic hybrid prosthesis (HP) infection, the surgery carries an extremely high risk. In this article, we present a case in which frozen elephant trunk was implanted with a second-time endovascular thoracic stent-graft repair who presented with an infection extending into the aortic root and the descending aorta.

The patient was a 48-year-old man with a history of ascending aorta aneurysm. A transthoracic echocardiogram (TTE) showed tricuspid aortic valve with severe aortic insufficiency, preserved ventricular function, and severe dilation of the aortic root. The patient had no physical features of Marfan syndrome or any other connective tissue disease. Computed tomography (CT) scan of the aorta showed a 56-mm diameter ascending aorta and 51-mm descending aorta.

A cardiac surgery was programmed in which Bentall-Bono procedure was performed with a 27-mm composite graft (St. Jude Medical, Minnesota, United States of America). Cerebral protection using moderate hypothermic circulatory arrest combined with selective cerebral perfusion was performed, and a total arch replacement was done with a frozen elephant technique using an E-Vita Open Plus graft (JOTEC® GmbH, Germany). The procedure was uneventful, and the patient was discharged home. The aortic valve pathology test showed granulomatous inflammation in the aortic wall and atherosclerosis phenomena with calcified plaques.

**Fig. 1:**
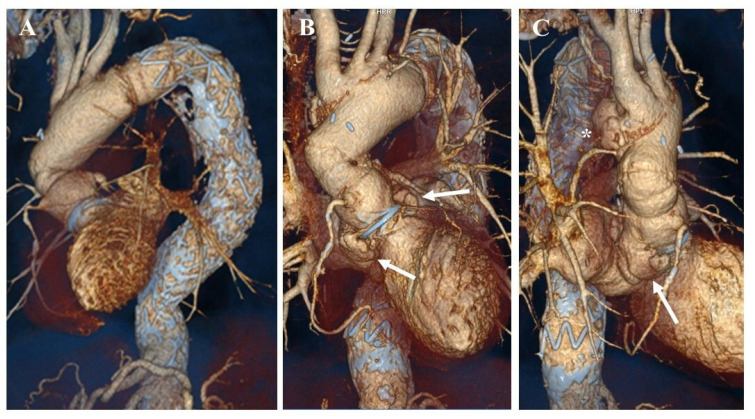
(A) Volume-rendering computed tomography (CT) angiography presenting the successful aortic root and ascending aorta replacement using the frozen elephant technique and the correction of descending aorta enlargement, by placing a two-piece combination stent. (B-C) Volume-rendering CT angiography images showing periprosthetic pseudoaneurysms (arrows) and a large pseudoaneurysm (*) involving the supra-aortic vessels.

Two years later, in a second-time procedure, the patient underwent thoracic endovascular aortic repair in which a percutaneous stent-graft implantation was performed using a two-piece combination of the Valiant Navion™ FreeFlo and CoveredSeal stent (36 × 150 mm). Posterior CT controls showed stability in aortic diameters, and the patient was asymptomatic (Figure 1).

**Fig. 2:**
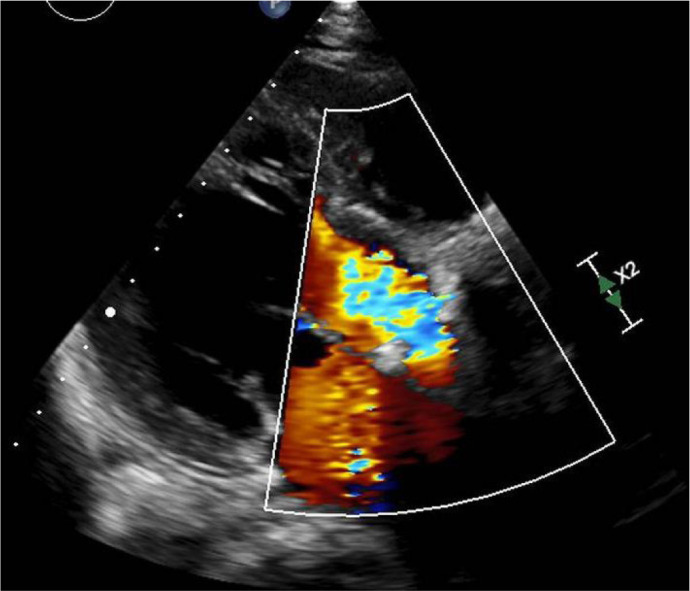
Transthoracic echocardiogram showing severe ventricular dilatation (left ventricular end-diastolic diameter of 68 mm) and massive aortic insufficiency because of ring suture dehiscence.

**Fig. 3:**
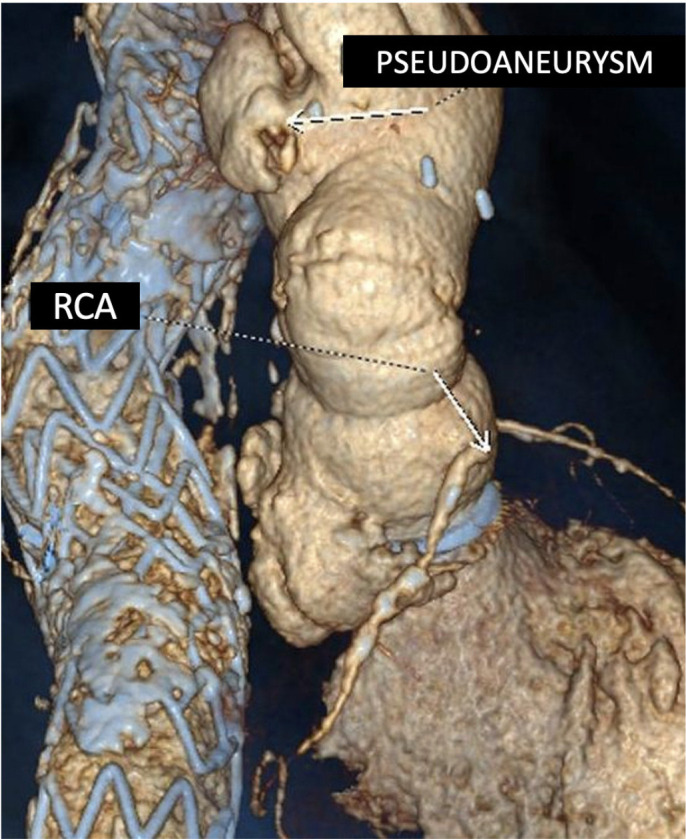
Computed tomography scan showing three aortic pseudoaneurysms: a proximal one extended above and below the prosthetic valve with wide communication with the left ventricle, a second pseudoaneurysm adjacent to the ostium of the reimplanted left coronary artery, and a posterior pseudoaneurysm that runs parallel to the aortic graft in the aortic arch without apparent communication with the previous pseudoaneurysms. RCA=right coronary artery.

Eight years later, the patient presented to the emergency department suffering an acute cardiogenic failure. No fever, vascular, or immunologic phenomena were observed. TTE showed an impaired ventricular function with severe ventricular dilatation and massive aortic insufficiency because of ring suture dehiscence (Figure 2). CT showed three aortic pseudoaneurysms, the proximal one extended above and below the prosthetic valve with wide communication with the left ventricle (Figure 3), a second pseudoaneurysm adjacent to the ostium of the implanted left coronary artery, and another posterior pseudoaneurysm that runs parallel to the aortic graft in the aortic arch. The patient underwent an emergency second redo surgery.

## TECHNIQUE

Cardiopulmonary bypass was established with femoral artery and vein cannulation. Median sternotomy was performed, and deep hypothermic circulatory arrest was achieved. Suture dehiscence of the supra-aortic anastomosis in the prosthetic tube was observed with infectious appearance. The aortic arch was opened under circulatory arrest with selective cerebral perfusion, and the three supra-aortic vessels were isolated. Proximally, the dehiscence of the left coronary ostium anastomosis was observed. Both native ostia were sectioned and isolated. The composite graft was explanted showing infectious material, however, the descending thoracic endoprosthesis appeared to be free of infection. Four-branched prosthetic graft (Vaskutek® 30 mm) was used to replace the arch and reconstruct the supra-aortic vessels. After completing the three anastomosis, systemic perfusion was restored, and rewarming was initiated. A biological valved tube was implanted (Magna Ease n. 23 + Vaskutek® Valsalva 30 mm) in the proximal aorta. Right coronary ostium was implanted directly in the proximal graft whereas left coronary ostium was implanted following a modified Hemi-Cabrol technique as one of the branches of the distal prosthetic graft. Finally, both grafts were sutured. Cardiopulmonary bypass time was 243 minutes, cross-clamping time was 238 minutes, and circulatory arrest time was 125 minutes.

**Fig. 4:**
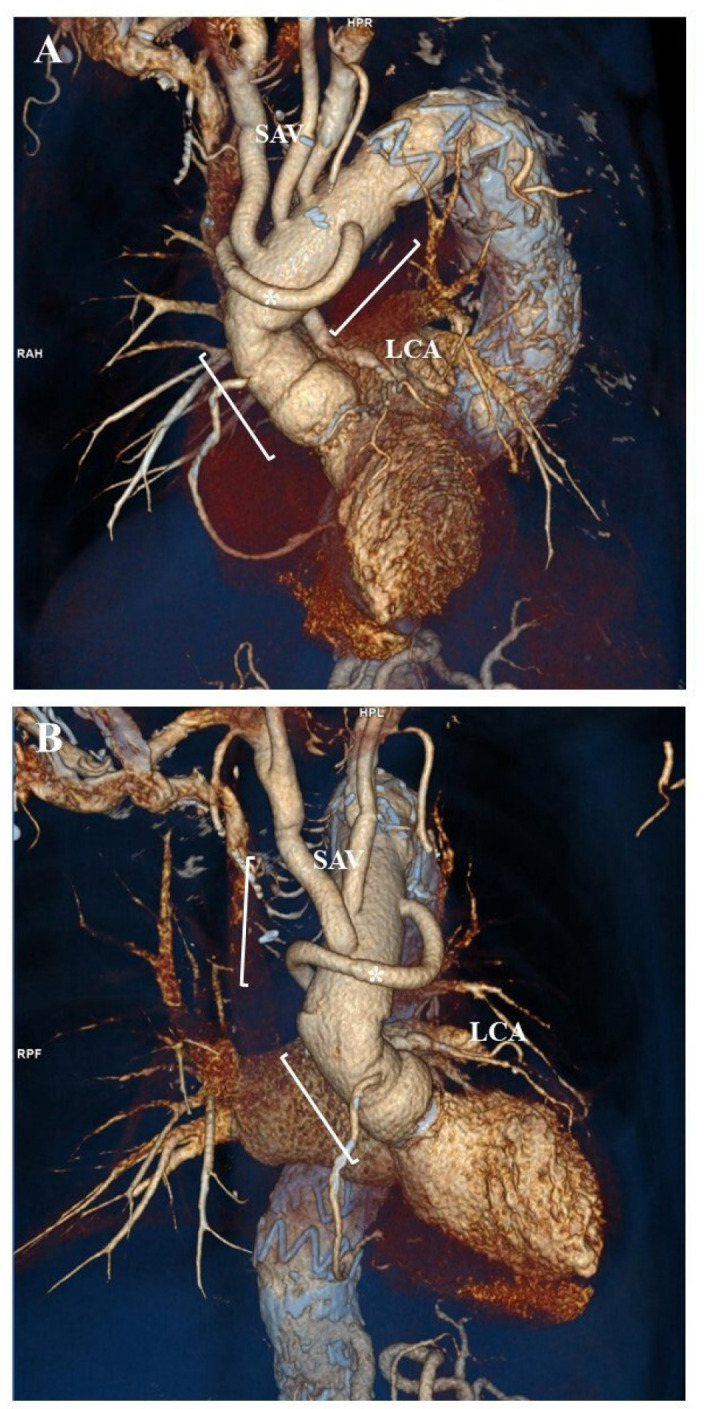
Volume-rendering computed tomography angiography after surgery showing the new composite aortic root graft and the four-branched prosthetic aortic arch graft with reimplantation of supra-aortic vessels (SAV) (square brackets). A third Dacron tube graft (*) is used to connect the left coronary artery (LCA) to the ascending aortic graft.

Right ventricular failure with severe systemic shock occurred after surgery, and extracorporeal membrane oxygenation was used as assistance during four postoperative days. Postoperative course was insidious but uneventful (Figure 4). The patient received an initial intravenous treatment with daptomycin, cloxacillin, and gentamicin followed by an association of amoxicillin and rifampicin after *Propionibacterium acnes* isolation in the graft culture.

Oral therapy with amoxicillin was established as a lifelong treatment as it is a low virulent microorganism but with a high resistance to antibiotics.

Since the surgical procedure four years ago, the patient has remained in New York Heart Association functional class I/IV without infectious complications or symptoms.

## DISCUSSION

Complete removal of the infected material is the optimal therapeutic approach to eradicate the infection. However, aggressive debridement and the explant the infected graft in an HP are associated with an increase in perioperative risks. Although isolated cases have been published^[2,3]^, HP infection has been a matter of little debate in the literature, and, accordingly, the management is not standardized.

Recently, Inaba et al.^[2]^ described a novel method for HP removal using a polyvinyl tube as the storage device and performed via median sternotomy. The polyvinyl water supply tube is cut and sterilized previously to be inserted to reach the distal endpoint of the stent graft. The authors described a technique suitable for the removal of all types of prosthesis and thoracic endovascular aortic repair stents, although it is not appropriate if there are obvious findings of the infection in the native aorta at the HP insertion site.

Risteski et al.^[3]^ performed a replacement of the aorta with a composite of three aortic homografts, and Nader et al.^[4]^ performed a replacement of the proximal compound of the HP accompanied by a complete debranching of the three supra-aortic vessels. In both cases, the distal stent portion of the HP was left *in situ*, following the same surgical strategy that we developed in our center. Although we should individualize every patient and situation, the total removal of an HP previously expanded and deployed within the aorta is technically challenging and carries inherent risk because of preexisting infection and the health status of the patient.

In addition, *P. acnes* is a rare but aggressive causal microorganism of endocarditis^[5]^, given its biofilm attachment to the prosthetic material; it has a slow growth and a subacute course, causing late infection with a challenging diagnosis^[6]^. Infective endocarditis caused by *P. acnes* was described to have an indolent presentation and to almost exclusively affect men with a prosthetic valve^[5]^. It has been also described as a rare cause of implant infections, most frequently due to direct contact with the patient’s skin^[4]^ or bacteraemia secondary to skin wounds. Penicillin, ceftriaxone, and rifampicin are the first-line antibiotics for *P. acnes*^[7]^.

In our case, *Propionibacterium* caused a cardiogenic shock related to massive periprosthetic regurgitation caused by prosthetic dehiscence eight years after the initial infection.

## CONCLUSION

We presented a case in which frozen elephant trunk was implanted with a second-time endovascular thoracic stent-graft repair who presented with a subacute graft infection caused by *P. acnes* extending into the aortic root and the descending aorta. The emergent redo-surgery consisted of the replacement of the graft by a biological valve tube accompanied by a complete debranching of the three supra-aortic vessels.

Although it is a technically challenging surgical approach, it is feasible and potentially successful with satisfactory medium-term results.
